# Dyslipidemia in Pulmonary Tuberculosis Patients Attending Nsawam Adoagyiri Municipal Hospital: Pre‐ and Post‐Anti‐Tuberculosis Treatment

**DOI:** 10.1002/hsr2.72175

**Published:** 2026-03-24

**Authors:** Prince Agyeman, Nixon Ntiri Somuah, Yussif Adams, Felix Abekah Botchway

**Affiliations:** ^1^ Department of Medical Laboratory Science KAAF University College Accra Ghana; ^2^ Department of Microbiology Nsawam‐Adoagyiri Municipal Hospital Nsawam Ghana; ^3^ Department of Medical Laboratory Science University for Development Studies Tamale Ghana; ^4^ Department of Medical Laboratory Science Accra Technical University Accra Ghana; ^5^ Accreditation and Affiliation Department Accra Technical University Accra Ghana

**Keywords:** anti‐tuberculosis, directly observed treatments (DOTS), dyslipidemia, Ghana, lipid profile, pulmonary tuberculosis

## Abstract

**Background:**

Tuberculosis (TB) remains a significant public health problem in Ghana, with high morbidity and mortality rates. Persons with TB are at risk of adverse cardiovascular disease with hyperlipidemia being a major risk factor. The standard treatment for pulmonary TB includes a combination of anti‐tuberculosis drugs such as Rifampicin, Isoniazid, pyrazinamide, and ethambutol which have been associated with changes in lipid profile that could increase the risk of cardiovascular disease. Despite the existence of the links between cholesterol and TB, it is not clear to what extent the treatment of the disease affects lipid indicators in patients with TB, particularly in Ghana. This study, therefore, sought to investigate any differences in the concentration of lipid profile in Pulmonary Tuberculosis patients in Nsawam Adoagyiri Municipality vis‐sa‐vis pre‐ and post‐anti‐tuberculosis treatment.

**Methods:**

A cross‐sectional survey involving 210 participants was conducted in Nsawam‐Adoagyiri Municipal Hospital, in the Eastern Region of Ghana. Data were analyzed using STATA version 17. Categorical variables were presented as numbers (percentages). Continuous variables with normal distribution were expressed as mean ± standard deviation. Inferential statistics were explored using paired *t*‐test. A *p* < 0.05 was considered statistically significant.

**Results:**

The overall dyslipidemia pre‐ and post‐tuberculosis treatment are 69.0% (145/210) and 77.6% (163/210) respectively. The mean Total Cholesterol and LDL increased significantly after the patient had completed the anti‐tuberculosis treatment (*p* < 0.001). However, there were no significant difference in the mean triglycerides (TG) and HDL levels before and after treatment.

**Conclusion:**

These findings suggest that dyslipidemia is quite prevalent among TB patients attending the Nsawam Government Hospital, which may have a negative effect on their health and general well‐being. There is a significant difference in the lipid profile results of the participants’ overall before and after the TB therapy. Anti‐TB treatment needs to develop more advanced recommendations that take patients' lipid levels into account in order to improve the outcomes for TB patients.

## 1 Introduction

Tuberculosis (TB) is one of the earliest diseases affecting humans since prehistoric times [1]. *Mycobacterium tuberculosis* is the disease's etiological agent. Tuberculosis is a contagious illness that typically affects the lungs (Pulmonary Tuberculosis). In Extra Pulmonary Tuberculosis, the infection affects other parts of the body like the stomach, lymph nodes, bones, meninges, skin, and genitourinary tract [2].

The pathogen is a bacillus that grows slowly and is known to spread through breathing. The bacterium is inhaled as droplets that travel through the respiratory system before being deposited in the alveoli [3, 4]. The signs and symptoms of TB include weight loss, a cough that lasts 2 weeks or more, pain in the chest with cough, coughing up blood or thick mucus, feeling weak or tired, diarrhea, night sweats, and hepatomegaly [2].

According to World Health Organization (2020) report, 10.6 million persons contracted TB globally. Between 2020 and 2021, the incidence rate of TB (new cases per 100,000 people per year) increased by 3.6%, reversing annual decreases of roughly 2% for the majority of the previous two decades [5]. In contrast to the global incidence rate of 130 per 100,000 population per year, 9 out of the 17 nations in West Africa in 2019 reported TB incidence rates of > 99 per 100,000 people per year [6]. The anticipated TB incidence rates for Liberia and Sierra Leone were 308 and 304 cases per 100,000 people, respectively. Nigeria, however, continues to have the highest TB burden in West Africa, with an absolute estimated incidence of 407,000 cases in 2016 [7].

According to Ghana's 2021 Global TB Report, an estimated 14,900 individuals died from TB in 2020, and 44,000 people were predicted to have contracted the disease [8]. Only 29%–34% of the anticipated incident TB cases in Ghana from 2015 to 2020 were found and reported to the National Tuberculosis Program (NTP). This means that every year, 66%–71% of incident TB cases go unreported [9].

Persons with TB are at risk of adverse cardiovascular disease (CVD) with hyperlipidemia being a major risk factor [10]. The standard treatment for pulmonary TB includes a combination of anti‐tuberculosis drugs such as Rifampicin (RIF), Isoniazid (INH), pyrazinamide (PZA), and ethambutol (EMB) which have been associated with changes in lipid profile that could increase the risk of cardiovascular disease [11, 12].

According to Metwally and Raheem [13], hypocholesterolaemia in TB is a consequence of the disease rather than being a risk factor and with regular consumption of Anti‐Tuberculosis Therapy (ATT). Akpovi, Gbaguidi [14] reported that the lipid indices total cholesterol, HDL, LDL, and TG were low at the time of diagnosis but normalized with recovery from TB. However, Sushilendu, Kumar [12] also reported that all the lipid parameters were significantly low at the time of diagnosis but there was a significant increase in the level of lipid profile parameters after the completion of ATT.

Despite the existence of the links between cholesterol and TB, it is not clear to what extent the treatment of the disease affects lipid indicators in patients with TB, particularly in Ghana. This study, therefore, sought to investigate any differences in the concentration of lipid profile in Pulmonary Tuberculosis patients in Nsawam Adoagyiri Municipality *vis‐sa‐vis* pre‐ and post‐anti‐tuberculosis treatment.

## 2 Methods

### 2.1 Study Design/Population

This study was a prospective cross‐sectional survey conducted in Nsawam‐Adoagyiri Municipal Hospital, in the Eastern Region of Ghana. A convenient sampling procedure was used to sample all newly diagnosed PTB patients who attended the Directly Observed Treatments (DOTS) center of Nsawam‐Adoagyiri Municipal Hospital on daily basis.

### 2.2 Study Location

The study was conducted in Nsawam‐Adoagyiri Municipal Hospital in the Eastern Region. According to the Country Coordinating Mechanism (CCM) of the Global Fund to Fight AIDS, TB, and Malaria report, the Eastern region has the highest prevalence of TB (33.4%) in Ghana [15]. The Nsawam‐Adoagyiri Municipal Hospital is one of the renowned district hospitals in the Eastern Region of Ghana, located at Nsawam, the capital of the Akuapim South Municipality. The Hospital which started as a 50‐bed capacity in 1928, has now been transformed into a 135‐bed capacity hospital. The Hospital provides a range of services including Internal Medicine, General Surgery, Obstetrics/Gynae Services, Dental Services, Pharmacy Services, Counselling & Testing (CT) for HIV/AIDS and it has Directly Observed Treatments (DOTS) for TB treatment and a general laboratory where bacterial confirmation of TB is carried out.

### 2.3 Sampling

The sample size of the study was calculated using the Cochran formula for quantitative sample size determination [n = Z^2^ (p×q)/d^2^]

where *n* = minimum sample size *z* = the critical probability value for a confidence level of 95% (1.96), *p* = estimated proportion (*p*) of tuberculosis, 136 per 100,000 [16], *q* = 1 − *p*, *d* = margin of error (0.05). *n* = 209 (minimum number to be enumerated).

An estimation of 10% loss out of 209 observations due to incomplete data is 21. Approximately 210 participants were recruited for the study which is more than the minimum number. All participants are newly diagnosed with PTB and are yet to start anti‐TB treatment (ATT). PTB patients who have been diagnosed with CVD and those on lipid‐lowering medications such as; antihypertensive drugs, antipsychotics, and so forth. were excluded from the study. Patients with treatment defaults were also excluded.

### 2.4 Data Collection Technique and Tools

At recruitment, a pre‐tested questionnaire was used to collect data on participants' socio‐demographic, lifestyle, and clinical characteristics such as age, sex, and comorbidities, including diabetes mellitus, hypertension, smoking, and alcohol use. The height (to the nearest cm) and weight (to the nearest Kg) were measured with a SECA body meter and a weighing balance (Hospibrand ZT‐120, England) respectively. The height was taken without the patient wearing footwear and the weight was measured with them wearing light clothing. Body mass index (BMI) was calculated as weight (kg) divided by the square of the height (m^2^) and classified based on W.H.O. classification; BMI < 18.5 for Underweight, 18.5–24.9 for Normal weight, 25.0–29.9 for Pre‐obesity/overweight, 30.0–34.9 for Obesity class I, 35.0–39.9 for Obesity class II, above 40 for Obesity class III [17].

The standard treatment regimen recommended by the W.H.O. and Center for Disease Control and Prevention (CDC) comprises an intensive phase of 2 months of administration of the first line anti‐TB drugs; rifampicin (RIF), isoniazid (INH), pyrazinamide (PZA) and ethambutol (EMB). This is followed by a 4‐month continuation phase of RIF and INH [18, 19]. The patients with positive MTB gene Xpert results were selected for serum lipid profile testing. 3 ml of fasting blood sample was collected from a peripheral vein of each PTB‐positive patient before the start of anti‐TB drug treatment (baseline). The serum of samples was separated after being span on the high gravitational force of centrifuge. Another 3 mL of blood was collected again on week 24 when the patient had completed the baseline drugs.

Lipid profile parameters such as total cholesterol (TC), high‐density lipoprotein (HDL), and triglyceride (TG) were measured enzymatically using DiaSys Respons 910 automated chemistry analyzer manufactured by DiaSys Diagnostic Systems in Germany. The comparison of diagnostic values of PTB patients was performed with normal values of these parameters. However, LDL was calculated using the traditional Friedewald's formula: LDL−C (mmol/L) = TC – HDL‐C – TG/2.2 [20]. Dyslipidemia were classified as TC ≥ 5.17 mmol/L, TG ≥ 1.69 mmol/L, LDL‐C ≥ 3.36 mmol/L, and HDL‐C < 1.03 mmol/L for men and < 1.3 mmol/L for women [21].

### 2.5 Ethical Consideration

Ethical clearance was obtained from the Ethical and Protocol Review Committee of the College of Health Sciences, University of Ghana Medical School, Korle‐Bu, Accra and consent was also sought from each participant of the study. Ethics identification number: (CHS‐Et/M.7‐P4.7/2023‐2024). Participants were informed about the purpose of the study, and written informed consent was obtained before data collection. Confidentiality and anonymity were strictly maintained.

### 2.6 Data Analysis

Data was entered into Microsoft Excel version 16 and exported to STATA version 17 for analysis. Categorical variables were presented as numbers (percent). Continuous variables with normal distribution were expressed as mean ± standard deviation. Inferential statistics were explored using paired *t*‐test. A *p* < 0.05 was considered statistically significant.

## 3 Results

### 3.1 Demographic Characteristics of Participants

Table 1 shows the distribution of demographic characteristics of the participants. Overall, 210 participants were enrolled in the study. The oldest participant was 75 years and the youngest was 20. The mean age was 43.9 ± 11.92. The majority of the participants were aged 30–49 years (59.1%). Male participants were higher in number (69.5%) compared to female participants. More than half (52.4%) of the subjects were single. The majority of the participants had primary education (35.7%), while few (9.5%) had completed College/University.

**Table 1:** Demographic characteristics of participants.

Variable	Categories	Frequency (*n* = 210)	Percent
Age groups	20–29	24	11.4
	30–49	124	59.1
	50–69	58	27.6
	≥ 70	4	1.9
Sex	Female	64	30.5
	Male	146	69.5
Marital status	Single	110	52.4
	Married	63	30.0
	Divorced	14	6.7
	Widowed	23	11.0
Education	Primary	75	35.7
	Junior High	60	28.6
	Senior High	55	26.2
	College/University	20	9.5
Employment	Unemployed	78	37.1
	Self‐employed	64	30.5
	Employed‐private sector	25	11.9
	Employed‐public sector	30	14.3
	Retired	13	6.2
Religion	Christian	127	60.5
	Muslim	69	32.9
	Traditional	14	6.7

### 3.2 Lifestyle and Clinical Characteristics of Participants

The lifestyle and clinical characteristics of participants are summarized in Table 2. The majority of the participants did not take in saturated and trans‐fats, low‐fiber, sugary foods and so forth, or perform exercise at all (101/210, 48.1%; 121/210, 57.6%) respectively. More than half of the participants did not smoke or drink alcohol (158/210, 75.2%; 119/210, 56.7%) respectively. Few participants had high blood pressure and high plasma sugar (72/210, 34.3%; 20/210, 9.5%) respectively. The majority of the participants had normal BMI (99/210, 47.1%). Few of the participants had TB‐related comorbidity (39/210, 18.6%).

**Table 2:** Lifestyle and clinical characteristics of participants.

Variable	Categories	Frequency (*n* = 210)	Percent
Frequency of performing exercise	Not at all	121	57.6
	Sometimes	68	32.4
	Always	21	10
Frequency of taking saturated and trans‐fats, low‐fiber foods, and so forth	Not at all	101	48.1
	Sometimes	73	34.8
	Always	36	17.1
Smoking	Yes	52	24.8
	No	158	75.2
Alcohol intake	Yes	91	43.3
	No	119	56.7
TB‐comorbidity	Yes	39	18.6
	No	171	81.4
High blood pressure	Yes	72	34.3
	No	138	65.7
High plasma sugar	Yes	20	9.5
	No	190	90.5
BMI (Kg/m^2^)			
Underweight	< 18.5	54	25.7
Normal	18.5–24.9	99	47.1
Pre‐obesity	25.0–29.9	40	19.1
Obese	≥ 30.0	17	8.1

### 3.3 The Mean Difference in Lipid Profile Among Participants

Table 3 shows the distribution of the mean difference in lipid profile among participants before and after anti‐tuberculosis treatment. The baseline Total Cholesterol (5.11 ± 1.048) and LDL (3.18 ± 0.921) increased significantly (*p* < 0.001) after the patient had completed the anti‐tuberculosis treatment (5.64 ± 1.458; 3.69 ± 1.324 respectively). However, there were no significant difference in the Triglycerides (TG) and HDL before and after treatment.

**Table 3:** Mean difference in lipid profile among pulmonary tuberculosis patients before anti‐tuberculosis treatment, and after 24 weeks of the treatment.

Lipid profile parameter (mmol/L)	Before treatment	After treatment	*p* value	95% conf. interval
TC	5.11 ± 1.048	5.64 ± 1.458	< 0.001	4.97−5.25* 5.44−5.83**
HDL	1.32 ± 0.342	1.37 ± 0.370	0.149	1.28−1.37* 1.38−1.42**
TG	1.33 ± 0.490	1.27 ± 0.611	0.256	1.27−1.40* 1.19−1.35**
LDL	3.18 ± 0.921	3.69 ± 1.324	< 0.001	3.06−3.31* 3.51−3.87**

Abbreviations: HDL, high‐density lipoprotein; LDL, low‐density lipoprotein; TC, total cholesterol; TG, triglyceride.

* Before treatment confidence interval.

** After treatment confidence interval.

### 3.4 Prevalence of Dyslipidemia Among Participants

**Figure 1:**
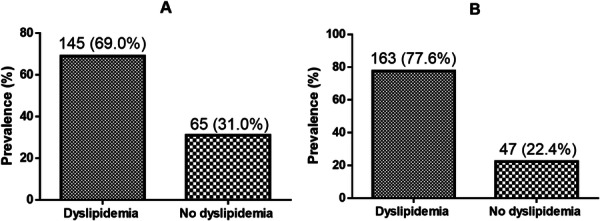
Prevalence of dyslipidemia among TB patients: (A) Pre‐treatment and (B) Post treatment.

Table 4 and Figure 1 shows the distribution of dyslipidemia among participants. The overall dyslipidemia pre‐ and post‐tuberculosis treatment are 69.0% and 77.6% respectively (Figure 1). The participants showed varying prevalence of dyslipidemia. The prevalence of elevated TC and LDL increased from 37.1% to 56.2% and 38.6% to 56.2% respectively after treatment. However, the prevalence of elevated TG and low HDL decreased from 22.9% to 16.7% and 30.0% to 24.8% respectively after anti‐TB treatment (Table 4).

**Table 4:** Dyslipidemia among TB patients pre and post anti‐tuberculosis treatment.

Lipid profile parameters	Frequency (*n* = 210)	Percentage (%)
Elevated TC		
Before treatment	78	37.1*
After treatment	118	56.2**
Low HDL		
Before treatment	63	30.0*
After treatment	52	24.8**
Elevated TG		
Before treatment	48	22.9*
After treatment	35	16.7**
Elevated LDL		
Before treatment	81	38.6*
After treatment	118	56.2**

Abbreviations: HDL, high‐density lipoprotein; LDL, low‐density lipoprotein; TC, total cholesterol; TG, triglyceride; VLDL, very Low‐density lipoprotein.

* Pre‐treatment prevalence

** Post‐treatment prevalence.

## 4 Discussion

Pulmonary Tuberculosis, a long‐term inflammatory disease of the lungs, is brought on by *M. tuberculosis*. Infectious illness deaths from TB are on the rise, and the disease is a global public health crisis. One‐third of people worldwide have MTB infection, according to the WHO [22]. Since the respiratory system is its primary method of transmission, TB is extremely contagious when it is active [23]. The phagocytic processes of cell magnetism, endocytosis, and exocytosis in macrophages all require cholesterol. Hence, the body's phagocytic function is compromised in cholesterol deprivation [24]. Lipids and their metabolites help reduce TB resistance via the immune system. Numerous disease disorders, particularly cardiovascular and diabetes mellitus, have been the subject of substantial research on lipids [25]. This study was performed to investigate any differences in the concentration of lipid profile in Pulmonary TB patients pre‐ and post‐anti‐tuberculosis treatment.

The overall prevalence of dyslipidemia in PTB before ATT initiation was estimated to be 69.0%. This however increased to 77.6% after patients had completed their treatment. The prevalence of dyslipidemia recorded after the completion of ATT was higher than the prevalence (73.9%) reported by Buasroung, Petnak [26]. Additionally, after therapy, the prevalence of high TC and LDL increased from 37.1% to 56.2% and 38.6% to 56.2%, respectively. The prevalence of hypercholesterolemia after the end of treatment in this study is higher than the prevalence (32.3% and 49%) observed in studies by Kanna [27] and Bonnefoy, Abidi [28]. The differences in the prevalence may be due to the different sample sizes or the geographical areas.

However, following anti‐TB therapy, the prevalence of increased TG and low HDL dropped from 22.9% to 16.7% and 30.0% to 24.8%, respectively. The findings of this study indicated that lipid concentration levels in ATT‐treated TB patients increased, which may have been due to an increase in CD4^+^ cells from the baseline to the sixth month follow‐up. The observed increase in lipid concentration levels among ATT‐treated TB patients may be explained by immune restoration and metabolic recovery occurring during effective anti‐tuberculosis therapy. Active tuberculosis is characterized by chronic inflammation, heightened catabolic state, and immune dysregulation, which are often associated with reduced serum lipid levels. During ATT, clearance of Mycobacterium tuberculosis leads to attenuation of systemic inflammation and recovery of immune function, including restoration of CD4⁺ T‐cell activity. CD4⁺ T cells play a critical role in coordinating host immune responses and are metabolically active cells that rely on cholesterol and other lipids for membrane synthesis, signaling platforms, and proliferation. As immune competence improves, lipid metabolism may normalize due to reduced inflammatory lipid consumption, improved hepatic lipid synthesis, and enhanced nutritional status [24, 25, 29]

Also, the improvement in nutritional condition, immunological function, and the clearing of circulating bacilli in the blood may all be contributing factors to the increase in lipid profile concentration in TB patients following ATT treatment [29, 30, 31].

According to the study's findings, there is a distinct difference between the lipid profile characteristics of TB patients before and after treatment. The participants' levels of TC and LDL were significantly elevated by the conclusion of the 24th week of treatment. At the end of the ATT, the levels of HDL also slightly rose. After the treatment, the levels of TG did, however, decline. The pattern of differences in the lipid parameters observed in this study is similar to the pattern of differences reported by Ahmed, Amjad [23] and Gebremicael et al., (2017). However, the findings of this study contradict the levels of TC, HDL and LDL reported in studies conducted in Turkey, Egypt and Benin by Deniz, Gumus [32], Metwally and Raheem [13], and Akpovi, Gbaguidi [14] respectively.

The TC levels (5.64 mmol/L) after ATT were significantly elevated compared to the TC levels (5.11) before the start of the anti‐tuberculosis treatment (*p* < 0.001). This finding is in agreement with a study conducted in Pakistan by Ahmed, Amjad [23] where a significant increase in TC levels was observed in TB patients after the completion of the ATT compared to TC levels before the start of treatment. The results of an experiment examining the effects of cholesterol supplementation during ATT revealed that it sped up the rate of culture conversion but had no effect on the symptoms [33].

According to other studies, fatty acids are used by MTB more frequently than carbohydrates. Therefore, during acute infections, MTB bacilli's consumption of cholesterol can reduce the host pool. Because it can also result in poor production rates and rapid catabolism during enervating illnesses, cholesterol levels should be kept at a minimum in patients with severe disabilities [34].

In the host cell, cholesterol is essential for MTB intracellular survival [35]. Studies supporting the claims demonstrated that phagosome maturation and autophagy blocks caused by pathogens could be overcome by cells losing their cholesterol levels [36]. In the claimed that dietary and serum cholesterol were related to the progression and results of active TB in humans, a study by Prabakaran, Efrem [37] was not able to predict the nature of the association.

The mean HDL before commencement of ATT increased from 1.32 mmol/L to 1.37 mmol/L after the treatment, which is higher than the levels reported by previous studies. Ahmed, Amjad [23] reported a mean HDL of 0.76 mmol/L before the start of ATT and 1.03 mmol/L at the end of ATT. However, the mean HDL reported in this study is lower than the mean HDL (1.42 to 1.54 mmol/L) reported by Kanna [27]. Early TB infection is accompanied by decreased HDL levels. Low HDL is a well‐known independent risk factor for poor cardiovascular outcomes, and it has also been demonstrated that this is true in people with TB infection, regardless of other risk factors [38]. Kanna [27] reported that regardless of changes in LDL, a rise in HDL is linked to a significant reduction in coronary heart disease (CHD)‐related mortality.

### 4.1 Limitations of the Study

This study is a prospective cross‐sectional study, and as such does not establish causality. Although baseline anthropometric and clinical characteristics such as BMI were assessed to characterize the study population, post‐treatment measurements of BMI and other metabolic syndrome components were not analyzed. This limits the ability to evaluate the dynamic relationship between dyslipidemia, weight changes, and metabolic syndrome following anti‐tuberculosis therapy. Given that nutritional recovery and weight gain commonly occur during TB treatment and may influence lipid metabolism, future longitudinal studies should incorporate repeated anthropometric, glycemic, and blood pressure assessments alongside lipid profiling which are warranted to provide a more comprehensive understanding of metabolic alterations associated with ATT.

## 5 Conclusions

It is concluded that newly diagnosed TB patients had desirable levels of cholesterol which however, increased significantly after the TB treatment. The results of the study showed a significant difference in the lipid profile results of the participants' before and after the TB therapy. According to the findings from this study, dyslipidemia is quite prevalent among TB patients attending the Nsawam Government Hospital, which may have a negative effect on their health and general well‐being. Anti‐TB treatment needs to develop more advanced recommendations that take patients' lipid levels into account in order to improve the outcomes for TB patients. The study's major goal is to aid researchers who need to do more in‐depth research on this subject by evaluating how anti‐TB medications affect cholesterol, HDL and LDL levels as well as supporting the development of effective TB treatment in Ghana.

## Author Contributions

Prince Agyeman and Felix Abekah Botchway perceived the study. Prince Agyeman and Felix Abekah Botchway developed the methods for investigation. Prince Agyeman and Nixon Ntiri Somuah were involved in data collection and analysis. Yussif Adams was involved in writing review and editing, and supervision. Felix Abekah Botchway was involved in writing review and editing, project administration, and supervision. All authors were involved in the development of the manuscript for submission. All authors have read and approved the final version of the manuscript Felix Abekah Botchway and Prince Agyemang had full access to all of the data in this study and takes complete responsibility for the integrity of the data and the accuracy of the data analysis.

## Funding

The authors received no specific funding for this work.

## Conflicts of Interest

The authors declare no conflicts of interest.

## Transparency Statement

The lead author Prince Agyeman, Felix Abekah Botchway affirms that this manuscript is an honest, accurate, and transparent account of the study being reported; that no important aspects of the study have been omitted; and that any discrepancies from the study as planned (and, if relevant, registered) have been explained.

## Data Availability

The data that support the findings of this study are available from the corresponding author upon reasonable request. The data used in this study are not publicly available due to confidentiality of information; however, the data is available from the corresponding author on a reasonable request basis.
